# Seasonal Shifts in Bacterial Community Responses to Phytoplankton-Derived Dissolved Organic Matter in the Western Antarctic Peninsula

**DOI:** 10.3389/fmicb.2017.02117

**Published:** 2017-11-03

**Authors:** Catherine M. Luria, Linda A. Amaral-Zettler, Hugh W. Ducklow, Daniel J. Repeta, Andrew L. Rhyne, Jeremy J. Rich

**Affiliations:** ^1^Department of Ecology and Evolutionary Biology, Brown University, Providence, RI, United States; ^2^Marine Biological Laboratory, Josephine Bay Paul Center, Woods Hole, MA, United States; ^3^Department of Earth, Environmental and Planetary Sciences, Brown University, Providence, RI, United States; ^4^NIOZ Royal Netherlands Institute for Sea Research, Utrecht University, Den Burg, Netherlands; ^5^Lamont-Doherty Earth Observatory, Columbia University, Palisades, NY, United States; ^6^Department of Marine Chemistry and Geochemistry, Woods Hole Oceanographic Institution, Woods Hole, MA, United States; ^7^Department of Biology, Marine Biology, and Environmental Science, Roger Williams University, Bristol, RI, United States; ^8^School of Marine Sciences, Darling Marine Center, University of Maine, Walpole, ME, United States

**Keywords:** 16S rRNA, amplicon sequencing, community assembly, bacterial succession, mesocosms, Collwelliaceae, *Polaribacter*, phytoplankton exudates

## Abstract

Bacterial consumption of dissolved organic matter (DOM) drives much of the movement of carbon through the oceanic food web and the global carbon cycle. Understanding complex interactions between bacteria and marine DOM remains an important challenge. We tested the hypothesis that bacterial growth and community succession would respond differently to DOM additions due to seasonal changes in phytoplankton abundance in the environment. Four mesocosm experiments were conducted that spanned the spring transitional period (August–December 2013) in surface waters of the Western Antarctic Peninsula (WAP). Each mesocosm consisted of nearshore surface seawater (50 L) incubated in the laboratory for 10 days. The addition of DOM, in the form of cell-free exudates extracted from *Thalassiosira weissflogii* diatom cultures led to changes in bacterial abundance, production, and community composition. The timing of each mesocosm experiment (i.e., late winter vs. late spring) influenced the magnitude and direction of bacterial changes. For example, the same DOM treatment applied at different times during the season resulted in different levels of bacterial production and different bacterial community composition. There was a mid-season shift from Collwelliaceae to *Polaribacter* having the greatest relative abundance after incubation. This shift corresponded to a modest but significant increase in the initial relative abundance of *Polaribacter* in the nearshore seawater used to set up experiments. This finding supports a new hypothesis that starting community composition, through priority effects, influenced the trajectory of community succession in response to DOM addition. As strong inter-annual variability and long-term climate change may shift the timing of WAP phytoplankton blooms, and the corresponding production of DOM exudates, this study suggests a mechanism by which different seasonal successional patterns in bacterial communities could occur.

## Introduction

Marine dissolved organic matter (DOM) represents a large reservoir of carbon that drives a considerable fraction of the oceanic food web (Hedges, 1992). Phytoplankton production is the dominant source of marine organic material, with up to 50% of algal production entering the DOM pool through a variety of mechanisms (Lampert, 1978; Fogg, 1983; Gobler et al., 1997). The resulting DOM pool is a complex mixture of thousands of organic compounds with varying degrees of lability. The labile DOM pool turns over rapidly, within hours to days, supporting the growth of heterotrophic bacteria. The semi-labile pool turns over more slowly, months to years, while the refractory pool turns over very slowly and accounts for the largest fraction of marine DOM (Carlson and Hansell, 2015). Despite ramifications for organic matter and nutrient cycling, the interplay between diverse bacterial assemblages and complex DOM pools is poorly understood.

Shifts in bacterial community composition are associated with phytoplankton blooms (Pinhassi et al., 2004; Teeling et al., 2012; Klindworth et al., 2014; Sperling et al., 2017), perhaps due to changes in DOM availability and phytoplankton composition of the blooms. Previous studies provide conflicting reports of generalist assemblages that can utilize a wide variety of substrates (Mou et al., 2007; Newton et al., 2010; Chronopoulou et al., 2015), as well as communities of specialists that are adapted to take advantage of only certain classes of DOM compounds and respond quickly to disturbance (Cottrell and Kirchman, 2000; Allison and Martiny, 2008; Nelson and Carlson, 2012; Sarmento and Gasol, 2012; Klindworth et al., 2014; Sharma et al., 2014). Nonetheless, some general trends based on broad taxonomic groups have emerged. For example, certain groups of bacteria (e.g., Flavobacteria and Rhodobacteraceae) tend to increase in abundance during phytoplankton blooms, while other groups such as *Candidatus* Pelagibacter appear better adapted to non-bloom conditions (Williams et al., 2013; Buchan et al., 2014; Voget et al., 2015).

The Western Antarctic Peninsula (WAP) system undergoes an extreme seasonal transition every spring, from almost total darkness to almost continuous sunlight, resulting in a synchronized cascade of environmental changes that culminates in intense phytoplankton blooms, supporting a highly productive food web (Venables et al., 2013). Bacterioplankton activity closely follows the annual phytoplankton cycle supporting the hypothesis that bacterial growth is largely driven by DOM availability (Kirchman et al., 2009; Ducklow et al., 2012; Kim and Ducklow, 2016). Luria et al. (2016) measured seasonal succession in the composition of free-living bacterial communities in nearshore waters of the WAP during a phytoplankton bloom. However, the relationship between phytoplankton-derived DOM and bacterial community succession has not been directly tested in the WAP. Furthermore, the WAP is subject to strong inter-annual variability in sea ice and upper water column dynamics, leading to variability in the timing and magnitude of phytoplankton blooms. Dramatic warming of the WAP region over the last 50 years has reduced sea ice extent and led to earlier sea ice retreat in the spring (Vaughan and Doake, 1996; Meredith and King, 2005; Stammerjohn et al., 2008; Thomas et al., 2009; Saba et al., 2014). It is not known how changes in the timing of sea ice retreat, and subsequently of phytoplankton blooms, may alter bacteria-DOM interactions and carbon cycling.

We conducted a series of DOM-addition mesocosm experiments that spanned the WAP spring transitional period to examine how pre-bloom bacterial communities react to changes in DOM concentration. Because many previous DOM-addition experiments have relied upon relatively simple compounds (e.g., glucose or amino acids), that may serve as poor analogs for the organic carbon utilized by marine bacteria (Straza et al., 2010; Ducklow et al., 2011), we used diatom exudates, a more complex substrate. Our goals were to assess how diatom exudates alter the bacterial community and how these alterations change depending on the timing of the experiment in relation to the progression of the season from winter to late spring. We assumed that DOM additions would stimulate bacterial production and change bacterial community composition and hypothesized that the magnitude of these DOM-driven changes would decrease as the season progressed in relation to phytoplankton biomass based on chlorophyll *a* (chl *a*) in the nearshore seawater. This hypothesis is based on previous studies in Antarctica indicating that bacterial communities become less limited by DOM as phytoplankton blooms develop (Ducklow, 2003). Therefore, bacterial communities are likely to be less responsive to DOM additions as phytoplankton blooms develop.

## Materials and Methods

### Environmental Monitoring of Source Water

Mesocosms were set up at Palmer Station, Antarctica, using seawater from the station’s intake, located at a depth of 6 m, 16 m from the shore of the station. This source water was monitored during the experimental period (August–December 2013) to determine any changes in environmental parameters that might influence bacterial community responses in mesocosm experiments. Samples for dissolved nutrients (phosphate, silicate, nitrite, and nitrate) and particulate organic carbon and nitrogen (POC and PN) were processed according to Palmer LTER standard protocols^1^. Briefly, nutrient samples were filtered through combusted 0.7-μm glass fiber filters (Whatman, GE Healthcare Life Sciences, Piscataway, NJ, United States) and frozen at -80°C until analysis on a SEAL AutoAnalyzer 3 (data available at doi: 10.6073/pasta/e893d71c5586769731875d49fde21b1d; Ducklow et al., 2016). Samples for DOC were filtered through combusted glass fiber filters and stored frozen until analyzed by high-temperature catalytic oxidation in a Shimadzu TOC-V, following previously described methods (Carlson et al., 2010). POC and PN samples were collected on combusted 0.7-μm glass fiber filters from 1 to 3 L seawater and were frozen at -80°C until analysis via combustion using a Perkin Elmer 2400 Series II CHNS/O Analyzer. Seawater intake chl *a* data are routinely collected through the LTER project (data available at doi: 10.6073/pasta/c2df461937789b5e53019dadcd29fc57; Schofield and Vernet, 2016). Bacterial production and abundance were determined as described below.

### DOM Preparation

Dissolved organic matter was collected from large-scale *Thalassiosira weissflogii* cultures (500 L total) (CCMP1051, National Center for Marine Algae and Microbiota, East Boothbay, ME, United States), a well-characterized marine diatom, grown in f/2 medium (Guillard, 1975). The cultures were grown and harvested in Rhode Island, and the resulting DOM exudates were transported to Antarctica as dried residues. To grow the cultures, seawater for the f/2 medium was drawn from Narragansett Bay and was first passed through 1-μm filters prior to chlorination at 25 ppm for 4 days, followed by passage through successive granulated carbon and 1-μm filters and a 150 W UV sterilizer at a rate of 80 L min^-1^ with a final pasteurization at 85°C just prior to use. After cooling, seawater was amended with 0.02% v/v each of f/2 “Solution A” and “Solution B” (Fritz Aquatics, Mesquite, TX, United States) and 0.003 g L^-1^ sodium metasilicate (Na_2_SiO_3_). Axenic *T. weissflogii* cultures (100 L each) were grown in custom, acid-washed polycarbonate containers under 34-watt T8 cool white fluorescent lamps (Philips, Andover, MA, United States). Air enriched with 1800–2000 ppm CO_2_ and passed through 0.2 μm PTFE filter capsules (Sigma–Aldrich, St. Louis, MO, United States) was bubbled through the containers. The cultures were harvested at 7 days when they had developed dense growth but were still in the exponential growth phase. To create an f/2 “control,” the same volume of f/2 media was treated identically, but was not inoculated with *T. weissflogii*. This control was to account for any DOM in the f/2-amended seawater itself.

Cells and particulate matter were removed from *T. weissflogii* cultures via peristaltic pump-driven serial filtration through combusted GF/F filters (Whatman, GE Healthcare Life Sciences, Piscataway, NJ, United States) and acid-washed 0.2 μm polyethersulfone membrane filter capsules (Whatman, GE Healthcare Life Sciences, Piscataway, NJ, United States) using acid-washed platinum-cured silicon tubing (Masteflex, Cole Parmer, Vernon Hills, IL, United States). The filtrate was collected in clean acid-washed polycarbonate containers, acidified by adding sufficient hydrochloric acid to lower the pH to ∼2–3. Solid phase extraction of hydrophilic DOM was performed by pumping the filtrate through C18 columns (Supelco, Discovery C-18; 10 g) at a rate of 50 ml min^-1^. The columns were prepared by washing with 100 ml methanol followed by 200 ml of ultra high purity water. DOM was recovered by flushing the columns with 100 ml of methanol. The DOM solution was gently warmed to 35°C and was concentrated by vacuum evaporation followed by flushing with nitrogen gas as needed, leaving a dried residue suitable for transport. Prior to mesocosm experiments, the dried DOM was re-dissolved in a 0.2 μm-filtered 10 mM sodium hydroxide solution and added to DOM treated mesocosms to a final concentration of 20 ± 4 μmol L^-1^ added DOC, based on difference between DOM+ and controls in August–October experiments (Supplementary Table 1).

### Mesocosm Experiments

Mesocosm experiments were conducted in August, September, October, and December 2013 at Palmer Station by filling acid-washed 50 L polycarbonate carboys with seawater from the station’s intake. The filled carboys were divided into two treatments: (1) control (no additions, *n* = 3) and (2) +DOM (carboys that received diatom exudates; *n* = 3). An additional f/2 control treatment (uninoculated growth media, *n* = 3) was included in the October experiment only. The DOC concentration in the f/2 control was not any different than controls without any added DOC. As such, the f/2 control served as an additional no DOC addition control. The mesocosms were incubated for 10 days at 0°C, except in the August experiment when mesocosms were incubated at 3°C. All experiments were conducted under a low-level of continuous light, 1 × 10^14^ quanta cm^-2^ s^-1^, as measured inside the carboys with a hand-held radiometer (Biospherical Instruments, Inc.).

Chlorophyll a (chl *a*) and phaeopigment, dissolved inorganic nutrients (phosphate, silicate, nitrite, and nitrate), and bacterial production (^3^H-leucine incorporation rates) were assessed every 48 h for 10 days as previously described (Luria et al., 2016). Bacterial abundance samples were also collected every 48 h and were analyzed by flow cytometry with SYBR^®^ Green I nucleic acid staining (Invitrogen, Carlsbad, CA, United States) on a Guava easyCyte flow cytometer (EMD Millipore, Billerica, MA, United States). Samples for bacterial community composition were collected on days 0, 6, and 10 by filtering ∼2 L water through successive 3.0 μm polycarbonate and 0.22 μm polyethersulfone (EMD Millipore, Billerica, MA, United States) filters. Filters were flash-frozen with liquid N_2_ and stored at -80°C until further processing. Samples for POC and PN were collected on days 0 and 10 and DOC on days 0, 6, and 10 and analyzed as described above. POC/PN from day 10 in December were lost due to an instrument malfunction. All sampling took place at incubation temperatures.

### 16S rRNA Gene Amplicon Sequence Library Generation

Sequence libraries were generated as in Eren et al. (2013), with modifications as described in Luria et al. (2016). Briefly, DNA was extracted from cells captured on the 0.22 μm pore size filters from mesocosm experiments [i.e., “free-living” bacterioplankton or any bacteria attached to micro-particles (Busch et al., 2017)] using a DNeasy Plant Mini Kit (Qiagen, Valencia, CA, United States) with an additional bead-beating step. For each DNA sample, the V6 hypervariable region of the 16S rRNA gene was amplified by polymerase chain reaction with custom fusion primers that contained Illumina adaptors and inline barcodes (forward primer) or dedicated indices (reverse primer) (Eren et al., 2013). Size-selected PCR products were quantified and pooled in equimolar amounts prior to sequencing on one lane of an Illumina HiSeq 1000 cycle paired-end run.

Low-quality sequences were filtered from the resulting data by discarding reads without 100% consensus between forward and reverse paired-end sequencing reads (Eren et al., 2013), resulting in more than 16 million sequences across 81 mesocosm samples. OTUs were clustered using Qiime (v 1.9.1; Caporaso et al., 2010) with open reference OTU picking with the default UCLUST method (Edgar, 2010), a minimum cluster size of 2, and a 97% similarity threshold and were assigned Greengenes taxonomy (version 13_8; McDonald et al., 2012). After removing OTUs classified as chloroplasts, rarefied libraries were produced by randomly down-sampling to the smallest library size of 64271 sequences spread among 14751 OTUs. All of our sequence data are MIMARKS-compliant (Supplementary Data Sheet 1) (Yilmaz et al., 2011) and have been deposited in the NCBI Sequence Read Archive under accession number SRP091049 and Bioproject PRJNA344476.

### Data Analysis

To separate the effects of incubation day in an experiment (i.e., days into incubation period), DOM+ treatment or control, and any differences between experiments on univariate bacterial (i.e., cell abundance or production) parameters, we performed ANOVA on nested linear models using the lm and anova functions in R (Chambers, 1992; Venables and Ripley, 2002; R Development Core Team, 2008). Two linear models are nested if one (the restricted model) is obtained from the other (the full model) by removing a term from the full model. An ANOVA performed on such nested models provides the equivalent of the *F* test for goodness of fit and indicates whether individual terms improve the model. This provided a statistical test of our hypothesis as the season progressed.

The number of OTUs observed in each library, Shannon’s diversity, and Pielou’s evenness, were calculated using the BiodiversityR package in R (Kindt and Coe, 2005). Differences in overall bacterial community composition between samples were visualized with non-metric multidimensional scaling (NMDS) based on Bray–Curtis similarity using the metaMDS function in the vegan R package (Oksanen et al., 2015). Co-occurrence network analyses based on Pearson’s correlations were used to examine relationships among OTUs and external variables (i.e., experimental treatments and bacterial production). Networks were generated using the Cytoscape CoNet plugin version 1.1.1.beta and were visualized in Cytoscape version 3.4.0 (Shannon et al., 2003; Faust et al., 2012). The R code used for all data analysis and figure production can be accessed at https://github.com/cmluria/DOM.

## Results

**FIGURE 1:**
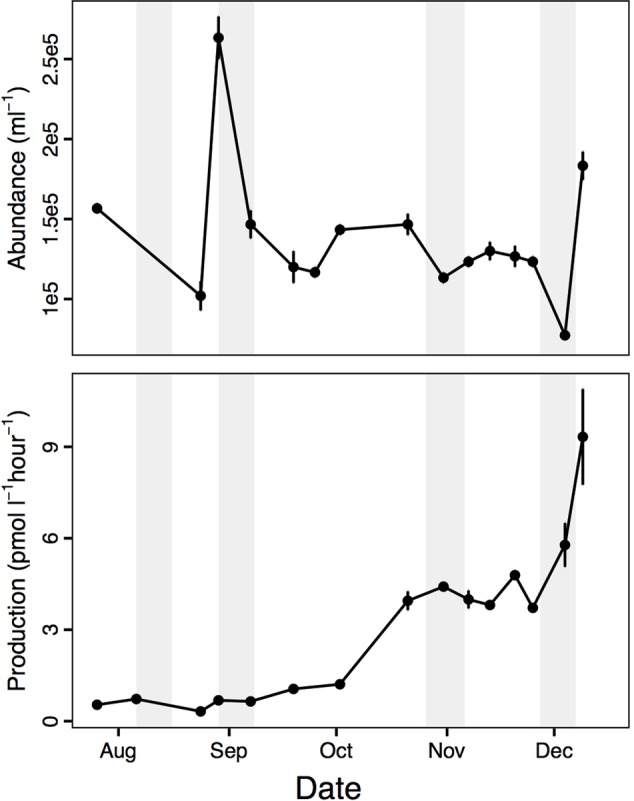
Bacterial abundance and leucine incorporation rate (production) in near shore surface water that was used in the dissolved organic matter (DOM) addition mesocosm experiments, August – December 2013 (average ± standard deviation, *n* = 3). Shaded bars indicate the timing of the four DOM addition experiments.

Our experiments spanned the winter-to-late spring period (August–December 2013), which was characterized by relatively constant environmental conditions (Supplementary Figure 1) compared to the onset of an intense phytoplankton bloom in summer, January 2014 (Luria et al., 2016). During the winter-to-late spring period, there was a slight uptick in leucine incorporation rates (hereafter bacterial production) in October and again in December (**Figure 1**). These changes in bacterial production corresponded to modest spikes in chl *a* concentration, suggesting a slight spring bloom (Supplementary Figure 1). To put these changes in context, peak chl *a* during the January summer bloom was ∼20 μg L^-1^ and bacterial production was ∼120 pmol L^-1^ h^-1^ (Luria et al., 2016). Nevertheless, temporal changes were already underway in the environment during the winter-to-late spring period.

**FIGURE 2:**
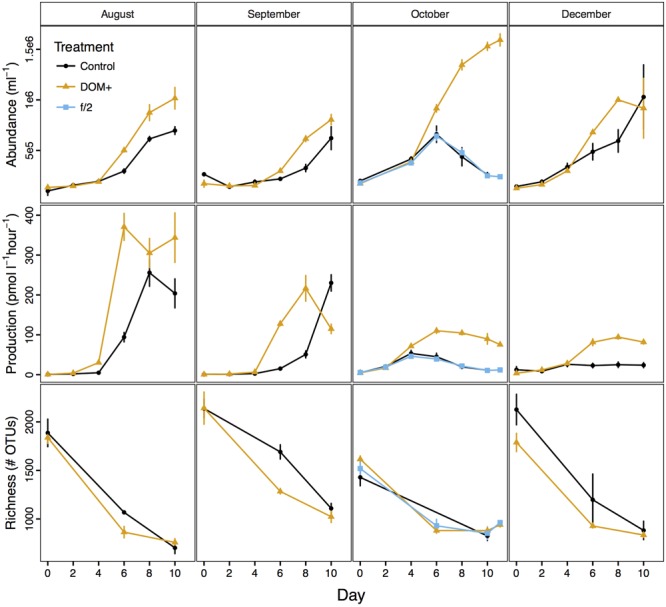
Bacterial abundance and production and observed OTU richness over the course of four mesocosm experiments (average ± standard deviation, *n* = 3).

Bacterial abundance and production increased over time in both control and DOM+ mesocosms, but increases were more rapid in the DOM+ mesocosms than in controls (**Figure 2**). As samples for bacterial production were not filtered, these measurements included both free-living and particle attached bacteria (and archaea). The DOC concentration in DOM+ or control mesocosms did not change significantly during incubations, indicating that most of the added or ambient DOC was non-labile (Supplementary Table 1). Changes in POC and PN provided further evidence for growth of bacteria during experiments (Supplementary Figure 2). Chl *a* and inorganic nutrients did not show consistent differences between treatments (Supplementary Figure 3). Based on comparisons of linear models, bacterial abundance was more significantly influenced by DOM treatment (*p* = 0.0002, *F* = 15.9) and incubation day (*p* = 6e-12, *F* = 76.5) than by what month the experiment was conducted (*p* = 0.01, *F* = 3.9) (Supplementary Table 2). Bacterial production was significantly influenced by all three factors (*p* < 0.005, *F* > 9). OTU richness of free-living bacteria declined in all experiments regardless of treatment (**Figure 2**). Evenness and Shannon diversity also declined in most mesocosms (Supplementary Figure 4). Incubation day and month of experiments had a significant effect on richness (*p* < 0.0001), while any effect of the DOM treatment was too small to pass the *p* < 0.05 cutoff (*p* = 0.08). Bacterial community composition in controls and the f/2 treatment were similar (Supplementary Figure 5).

**FIGURE 3:**
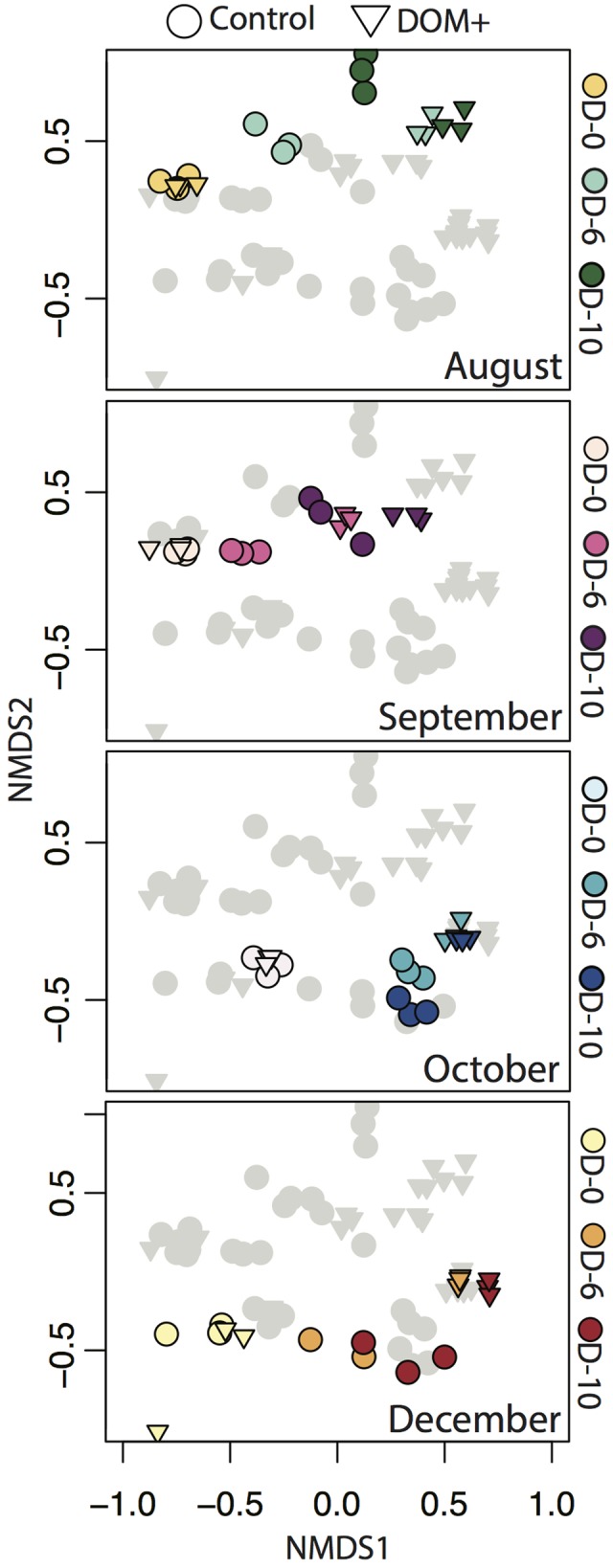
Non-metric multidimensional scaling (NMDS) of Bray–Curtis similarity indices based on OTU relative abundance across all four experiments. Each point corresponds to an individual mesocosm replicate (*n* = 3 per time point per treatment). All samples from all experiments are shown in each panel. Within each panel, a different experiment is highlighted with different colors corresponding to different sample days (0, 6, and 10), with the other experiments shown in gray. Control mesocosm communities are denoted by circles and DOM+ mesocosm communities are denoted by triangles.

Non-metric multidimensional scaling based on Bray–Curtis similarity demonstrated changes in overall free-living bacterial community composition across experiments (**Figure 3**). NMDS axis 1 reflected changes in community composition by incubation day. Changes in controls represent container effects, while changes in DOM+ treatments beyond controls reflect the influence of DOM addition. There were more rapid and greater changes in DOM+ treatments compared to controls. Axis 2 reflected differences in community composition between the start and end of the first two experiments (August/September) compared to the second two experiments (October/December). The grouping by August/September compared to October/December reflected differences in starting community composition between the two periods. The relative change in overall community composition, as reflected in the NMDS, was of similar magnitude across experiments.

**FIGURE 4:**
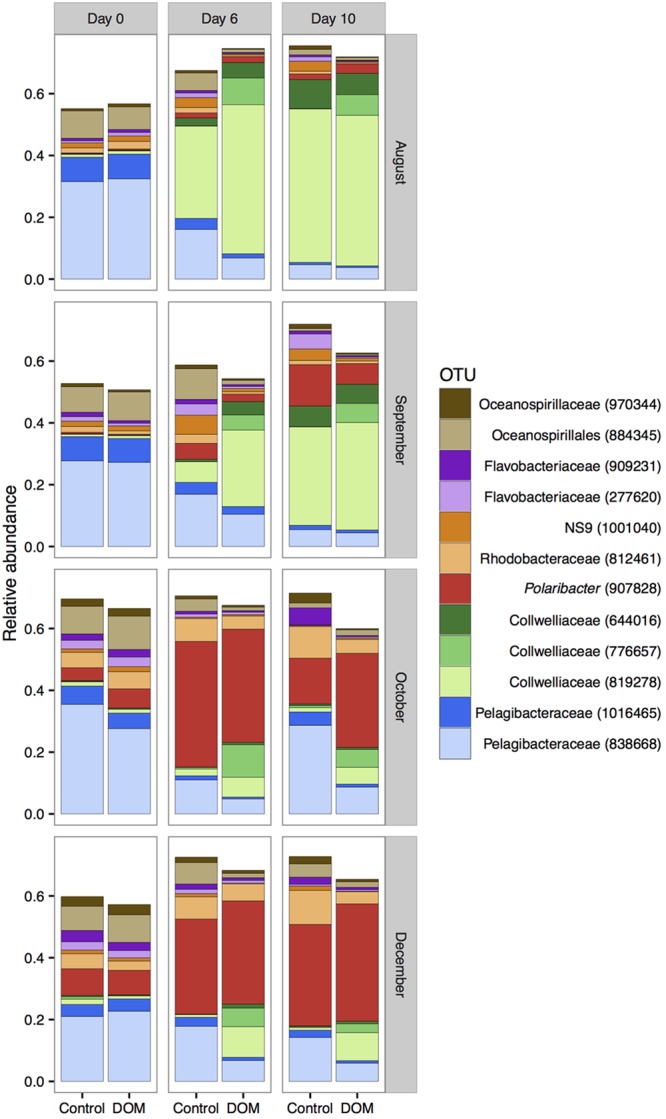
Changes in the mean relative abundance of the top 12 OTUs (by mean relative abundance) across all samples (*n* = 3). Note that the y-axis does not go up to 1 as the top 12 OTUs are shown instead of all OTUs.

The top 12 OTUs of free-living bacteria (by mean relative abundance across all samples) together represented 50–75% of all sequences. In all four experiments, OTUs classified as Pelagibacteraceae, Oceanospirillales, and SAR324 declined over time, while OTUs classified as Collwelliaceae and *Polaribacter* increased in relative abundance over time (**Figure 4**). Although the most abundant Collwelliaceae OTU (819278) displayed container effects, it had higher relative abundances in DOM+ mesocosms than controls on day 6 in August/September and on day 6 or 10 in October/December, indicating a response to DOM addition beyond container effects (**Figure 4** and Supplementary Figure 6). A different Collwelliaceae OTU (776657) only increased in DOM+ mesocosms and not in controls, indicating a consistent response to DOM addition as the season progressed (**Figure 4** and Supplementary Figure 6). The relative abundance of the most abundant Collwelliaceae OTU (819278) and another Collwelliaceae OTU (6644016) shifted between August/September and October/December. This shift corresponded to a smaller but significant *in situ* increase in the relative abundance of *Polaribacter* (907828) in October/December compared to August/September, suggesting that the higher starting abundance of *Polaribacter* (907828) ultimately led to its higher final abundance in October/December. Of the top 12 OTUs, 6 varied in relative abundance by 5% or more at one or more time points between the DOM+ and control communities (Supplementary Figure 6). Rhodobacteraceae (812461), Oceanospirillales (884345), and Pelagibacteraceae (838668) had similar or lower relative abundances in DOM+ mesocosms compared to controls. *Polaribacter* (907828) displayed both positive and negative effects between DOM+ and controls at different times, but it always showed an increase in relative abundance after the start of experiments, irrespective of treatment (**Figure 4**).

**FIGURE 5:**
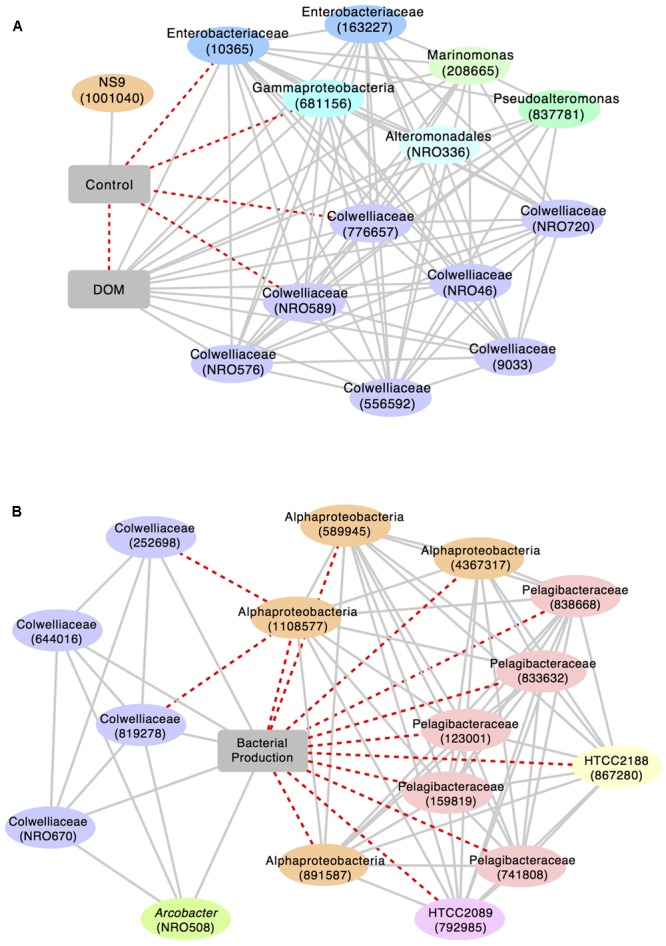
Sub-networks of highly correlated OTUs built around **(A)** treatment level (control vs. DOM+; *r* > 0.5) and **(B)** bacterial production (*r* > 0.7). Solid, gray lines represent positive correlations; dashed, red lines represent negative correlations. Closest taxonomic identification and reference number (in parentheses) are given for each OTU. “NRO” in the reference number indicates a new reference OTU, i.e., an OTU generated through Qiime’s open reference OTU picking pipeline from sequences that did not initially match against the Greengenes database.

We identified a sub-network of OTUs that were significantly associated with either the control group or the DOM treatment (*r* > 0.5; **Figure 5**). All of the OTUs that were positively correlated with DOM+ were classified as Gammaproteobacteria, including Collwelliaceae. Different OTUs identified as Collwelliaceae, reflected diversity within this family of bacteria. A few OTUs in this sub-network were negatively correlated with the control. A second sub-network linking OTUs to bacterial production showed that four Collwelliaceae OTUs correlated positively with bacterial production. Alphaproteobacteria OTUs, over half of them classified as Pelagibacteraceae, as well as two Gammaproteobacteria, HTCC2089 and HTCC2188, were negatively correlated with bacterial production (*r* > 0.7).

## Discussion

Phytoplankton blooms and the resulting release of labile DOM are thought to be a major driver of marine bacterial community composition. Observational studies have shown that bacterial community composition and activity vary during phytoplankton blooms (Pinhassi and Hagström, 2000; Fandino et al., 2001; West et al., 2008; Tada et al., 2011; Teeling et al., 2012; Klindworth et al., 2014; Wemheuer et al., 2015). Similarly, the composition of DOM has been shown to change during phytoplankton blooms (Zhang et al., 2015; Sperling et al., 2017). This body of work suggests that bacterial succession is coupled to different stages of DOM decomposition through changes in relative abundance of bacterial groups with different metabolic strategies and substrate preferences (Cottrell and Kirchman, 2000; Alonso-Sáez and Gasol, 2007; Poretsky et al., 2010; Rinta-Kanto et al., 2012; Sarmento and Gasol, 2012; Teeling et al., 2012). This pattern has been cited as evidence in support of niche partitioning, which may relieve competition between taxa and help explain high microbial diversity (Hutchinson, 1957; Teeling et al., 2012; Alexander et al., 2015). Alternatively, some studies have found that changes in DOM supply only slightly affect bacterial community composition, suggesting that physiological responses of metabolically versatile bacteria may be a factor, in addition to niche partitioning (Kirchman et al., 2004; Rooney-Varga et al., 2005; Rink et al., 2007).

There is growing evidence that labile organic matter availability is a primary factor controlling bacterial growth and community composition in the Southern Ocean (Thingstad and Martinussen, 1991; Kirchman et al., 2009; Ducklow et al., 2012; Kim et al., 2014; Luria et al., 2016). However, directly testing the effects of phytoplankton-derived DOM on bacterial seasonal succession has proven challenging. Mesocosm experiments, like the one we conducted here, have traditionally relied on adding low molecular weight compounds like glucose or amino acids (Havskum et al., 2003; Allers et al., 2007; Gómez-Consarnau et al., 2012). For example, Ducklow et al. (2011) demonstrated that glucose enrichment of WAP seawater reduced bacterial diversity. Using more complex DOM substrates derived from phytoplankton cultures, several recent studies have shown that a wider range of bacterial taxa responds readily to phytoplankton, especially diatom-derived DOM, and that DOM originating from different phytoplankton species stimulates different bacterial phylogenetic groups (Romera-Castillo et al., 2011; Nelson and Carlson, 2012; Sarmento and Gasol, 2012). Conversely, Landa et al. (2014) and Sharma et al. (2014) found that varied natural DOM sources did not have differential effects on bacterial community composition despite variation in carbon quality and quantity. We selected the diatom *T. weissflogii* as a DOM source. Other species of *Thalassiosira* are widespread in the WAP region, suggesting that the DOM that we added could be a good analog for DOM exudates for part of the phytoplankton community. However, the quantity and quality of DOM exuded by phytoplankton vary with species and physiological state and DOM from a single source (one species in one growth phase) does not represent the entire spectrum of changes in DOM composition and supply that probably occur during a phytoplankton bloom (Becker et al., 2014; Landa et al., 2014). Similarly, the isolation technique that we used selects for hydrophobic DOM and does not efficiently concentrate very polar compounds. We assume that qualitatively our DOM extracts contained many of the same compounds or types of compounds produced by microbes in seawater. However, we recognize that the amounts and specific details of the compounds will be different. The concentration of DOC that we added is in the range of temporal variation at our study location. Background DOC based on deep-water samples is ∼40 μmol L^-1^ and nearshore surface water early in spring is around this concentration, consistent with the pattern that we observed in the environment during our study (Supplementary Figure 1). DOC increases and becomes more variable as the season progresses and phytoplankton blooms develop (average ± standard deviation of 51 ± 11 μmol L^-1^) (Ducklow, 2017), similar to other coastal polar locations (Kirchman et al., 2009).

In our experiments, temporal changes in controls followed those that occurred in DOM treatments, indicating container effects of increasing cell numbers of rapidly growing bacteria of certain taxonomic groups over others (Zobell, 1943; Ferguson et al., 1984; Eilers et al., 2000; Massana et al., 2001). *Polaribacter* are heterotrophs that target high molecular weight organic matter and increase rapidly during phytoplankton blooms in coastal Antarctic waters (Fernández-Gómez et al., 2013; Williams et al., 2013; Luria et al., 2016). The relative abundance of *Polaribacter* increased rapidly in our experiments, similar to other incubation experiments of coastal Antarctic waters (Massana et al., 2001; Landa et al., 2016). The specific *Polaribacter* OTU (907828) that increased in our experiments was also abundant in the environment, reaching peak relative abundance of 20–50% of sequences at our study location, depending on the year (Luria, 2016; Luria et al., 2016). The most abundant Collwelliaceae OTU (819278) in our experiments, showed strong increases in controls, but also consistent increases in DOM treatments over controls. A different Collwelliaceae OTU (810446) increased rapidly in relative abundance in the environment (up to ∼30% of sequences, depending on the year) during the primary phytoplankton bloom in our study area (Luria, 2016; Luria et al., 2016). Based on flow cytometry of bacterioplankton in our study area, Bowman et al. (2017) found an increase in the proportion of high nucleic acid bacteria compared to low nucleic acid bacteria during a phytoplankton bloom, indicating a physiological shift in bacterial communities in addition to taxonomic changes. *Colwellia* spp. are commonly isolated psychrophiles from polar sea-ice or deep-sea environments and model organisms of psychrophily, reflecting their capacity for rapid growth in cold environments (Deming et al., 1988; Bowman et al., 1997; Methé et al., 2005). Based on culture-independent techniques, *Colwellia* spp. had a high relative abundance in deep Antarctic coastal waters, associated with sinking *Phaeocystis* particles (Delmont et al., 2014). Conditions conducive to rapid growth of bacterioplankton occur in Antarctic coastal waters and these types of bacteria were favored in our experiments.

In our experiments, we observed differences in bacterial production and community composition that corresponded to different times during the winter-to-late spring period. Bacterial production in the mesocosms decreased from August/September to October/December, indicating the study period was split into two phases, rather than changing constantly in one direction or another, as we initially hypothesized. Subtle changes in the environment that occurred before our October experiment, as reflected in chl *a* and bacterial production, might be related to the shift that we observed. Rather than a decrease in magnitude of overall changes in community composition between the two periods, we observed a shift in community composition from Collwelliaceae OTUs (819278 and 6644016) in August/September to *Polaribacter* (907828) in October/December. This shift appears to be caused by changes in the relative abundance of *Polaribacter* (907828) in the starting seawater, which seemed to enable *Polaribacter* (907828) to outcompete the two Collwelliaceae OTUs (819278 and 6644016) in the October/December experiments. Meanwhile, another Collwelliaceae OTU (776657) did not appear to be affected by the increase in relative abundance of *Polaribacter* (907828). The factors that governed the negative relationship between OTUs are not clear but could be related to competitive interactions, direct antagonism, or differential top-down control (Fuhrman, 2009). Our study suggests that diversity within the Collwelliaceae could have functional implications for interactions among other taxa of bacterioplankton during phytoplankton bloom periods.

Despite longstanding debate about the role of temperature in regulating bacterial production (Pomeroy and Wiebe, 2001; Kirchman et al., 2009), a decade-scale study found that the relationship between bacterial production and temperature varied from year to year and was even negative in some years (Ducklow et al., 2012). In our study, although August mesocosms were incubated at 3°C, changes in community composition between August and September were similar. Ducklow et al. (1999) found no effect of increasing temperature from 2° to 4°C on bacterial production in incubations of Ross Sea water. Differing bacterial mortality rates in the initial source water might have been a factor. Predation in WAP waters has been shown to increase exponentially with temperature and during natural or simulated phytoplankton blooms (Bird and Karl, 1991; Duarte et al., 2005; Garzio et al., 2013). Brum et al. (2016) found that viral abundance and lysogeny increased during the spring-to-summer transition in our study region. Bowman et al. (2017) further suggested the importance of top-down controls in structuring bacterial communities in Antarctic coastal waters in addition to bottom-up controls.

Our initial hypothesis of change in bacterial community responses as the season progressed was grounded in the niche model of community assembly wherein similar environmental conditions (e.g., DOM enrichment) select for the same or similar species from a diverse initial species pool, producing communities with similar structures (Fuhrman et al., 2006). This contrasts with the neutral model of community assembly in which stochastic forces, including growth, dispersal, and mortality are dominant forces in community assembly (Hubbell, 2001). While the differences we observed may be governed by factors that we did not consider (i.e., predation), our findings could be interpreted as priority effects in which variation in the initial relative abundance of species alters final community structure in addition to environmental filtering (Drake, 1990; Chase, 2007; Fukami and Nakajima, 2011; Nemergut et al., 2013). Although priority effects have been demonstrated for sequential colonization of a site by microbes, the application of this concept to slight numerical advantages in a complex initial inoculum is less clear (Warren et al., 2003; Jiang and Patel, 2008; Peay et al., 2010). Strong inter-annual and long-term climate change drives variation in WAP sea ice extent and duration and hence the timing and intensity of phytoplankton blooms (Smith et al., 2008). Subtle changes in bacterial community composition during spring set the stage for the ultimate trajectory of bacterial succession upon DOM addition.

## Author Contributions

CL, LA-Z, HD, and JR designed and conducted the study. LA-Z and JR provided sequence data for the study. AR and CL cultivated diatoms and DR and CL collected DOM. CL analyzed data and wrote the manuscript with guidance from JR, LA-Z, and HD. All authors approved the final version of the manuscript.

## Conflict of Interest Statement

The authors declare that the research was conducted in the absence of any commercial or financial relationships that could be construed as a potential conflict of interest.

## Footnotes

1 http://oceaninformatics.ucsd.edu/datazoo/data/pallter/datasets
