# Supplementary material for: Seasonal Shifts in Bacterial Community Responses to Phytoplankton-Derived Dissolved Organic Matter in the Western Antarctic Peninsula
Source: Front Microbiol. 2017 Nov 3;8:2117. doi: 10.3389/fmicb.2017.02117 (PMC5675858; doi:10.3389/fmicb.2017.02117)
Supplement: Supplementary file 2 [file Presentation_1.PDF]

DOC results (Supplemental Table 1). The DOC concentration was higher in DOM+ compared to controls, based on non-overlapping average ± standard deviations, except the December samples. The December samples had anomalously high concentrations in the day 0 controls (i.e., >70 μmol L^{-1}), and were thus excluded from further interpretation. The difference in DOC concentration between DOM+ and controls indicates the concentration of DOC exudates that were added to mesocosms, with the exception of December. Across the August-October experiments, we added an average of 20 ± 4 μmol L^{-1} DOC to DOM+ treatments (averaging the standard deviation across Aug-Oct experiments). We assumed that DOC added in the December experiment was not significantly different than that added in Aug-Oct experiments, as DOM extracts were treated the same for each experiment. Although average DOC concentration in DOM+ was somewhat lower in Day 10 (August) samples compared to Day 0 and 6, this difference is not significant based on overlapping average ± standard deviations. In controls in August or DOM+ and controls in September and October, there were not significant decreases in DOC concentration during incubations, indicating that the majority of DOC added in DOM+ treatments or naturally present in controls was non-labile. The concentration of DOC in the f/2 control was not any different than controls, indicating that the f/2 treatment served as an additional no DOC addition control.

Supplemental Table 1. Concentration of DOC (μmol C L^{-1}) in mesocosm experiments. The average ± standard deviation and number of replicate mesocosms in which DOC was measured (n)[a] are shown.

	DOM+	n	Control	n	Difference[b]
**August**					
Day 0	71.6 ± 9.0	3	45.8 ± 7.9	3	25.7 ± 8.4
Day 6	61.9 ± 2.2	2	45.3 ± 4.2	3	16.7 ± 3.2
Day 10	55.9 ± 5.3	3	47.5 ± 5.7	3	8.4 ± 5.5
**September**					
Day 0	54.8 ± 2.2	3	41.8 ± 0.6	2	13.0 ± 1.4
Day 6	63.6 ± 1.9	2	48.6 ± 10.4	2	15.0 ± 6.2
Day 10	64.5	1	40.7	1	23.7
**October**					
Day 0	63.8 ± 2.8	2	41.8 ± 0.6	2	22.0 ± 3.0
Day 6	65.0 ± 6.6	3	ND[c]	0	ND
Day 10	59.9 ± 6.2	2	35.3 ± 6.8	2	24.6 ± 6.5
			**f/2**		
Day 0			38.6 ± 5.3	3	
Day 6			41.1 ± 5.5	3	
Day 10			42.9 ± 5.3	3	
**December**					
Day 0	61.6 ± 3.7	3	77.8 ± 40.1	3	-16.3 ± 21.9
Day 6	35.6 ± 5.7	2	66.0 ± 3.1	2	-30.4 ± 4.4
Day 10	34.0 ± 0.3	3	27.9 ± 17.0	2	6.1 ± 8.6

[a]The number of carboys in which DOC was measured was not always n=3 as some sample labels fell off of frozen sample bottles during shipping.
[b]The difference between average DOC concentration in DOM+ and control treatments is shown, along with the average standard deviation across DOM+ and control treatments.
[c]ND, no data.

Supplemental Table 2. ANOVA of nested linear regressions. Each row represents an ANOVA test comparing two linear models, a restricted model with two variables compared to the full model with three variables (month, day, and treatment). The difference in sum of squares, *F* and *P* values reflect the improved fit of the full model over the restricted model.

Dependent variable	Restricted model	Full model - Independent variable tested	Residual degrees of freedom	Difference sum of squares	*F*	*P*
Abundance	Month + Day	Treatment	55	-1e12	15.9	0.0002
Abundance	Month + Treatment	Day	55	-5.0e12	76.5	6.23e-12
Abundance	Day + Treatment	Month	57	-7.5e11	3.9	0.014
Production	Month + Day	Treatment	66	-5.0e4	9.2	0.003
Production	Month + Treatment	Day	66	-2.2e5	39.5	3.1e-8
Production	Day + Treatment	Month	68	-2.2e5	13.3	7e-7
OTU richness	Month + Day	Treatment	66	-1.4e5	3.2	0.08
OTU richness	Month + Treatment	Day	66	-1.2e7	275	2.2e-16
OTU richness	Day + Treatment	Month	68	-1.7e6	12.9	1e-6

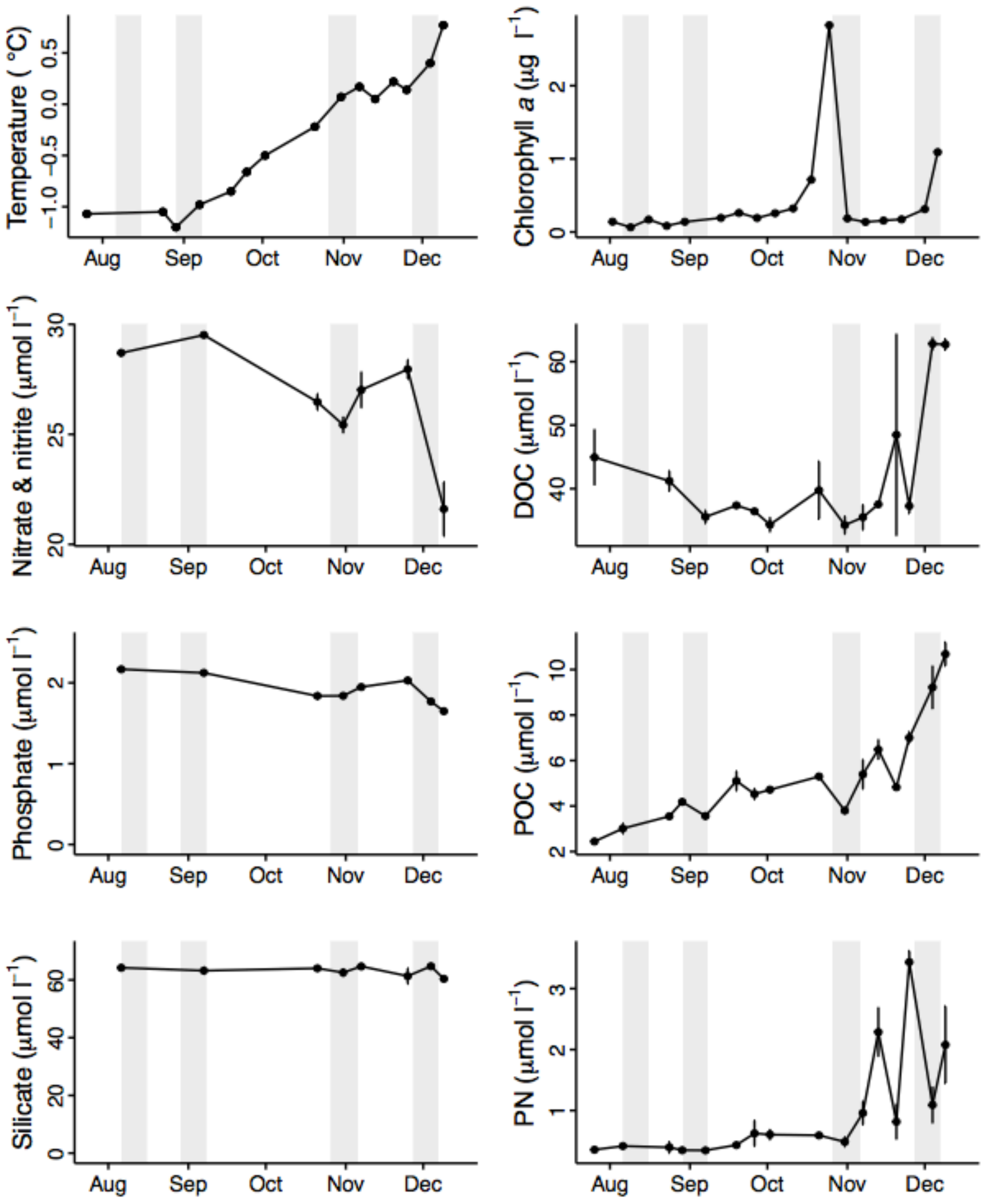

Supplemental Figure 1. Temperature, inorganic nutrients (nitrate/nitrite, phosphate, silicate), chl *a*, DOC, POC, and PN in nearshore water used in the DOM addition experiments, August – December 2013 (average ± standard deviation, n=3). The timing of the four DOM addition experiments is indicated by shaded bars. Note that the October experiment was started on October 26 and ended on November 5. During our experimental period, temperatures increased steadily from -1.2 to 0.8 °C. Inorganic nutrients (nitrate and nitrite, phosphate, silicate) were somewhat lower in October and December, but were never depleted. DOC was slightly lower than the deep-water signature (i.e., ~40 µM) during sample times in Sept-Nov, perhaps reflecting ice melt during this time. POC increased from about 2 to 10 µmol L^{-1} in the environment, but this increase is minor relative to the concentration of 80 µmol L^{-1} recorded during the January phytoplankton bloom.

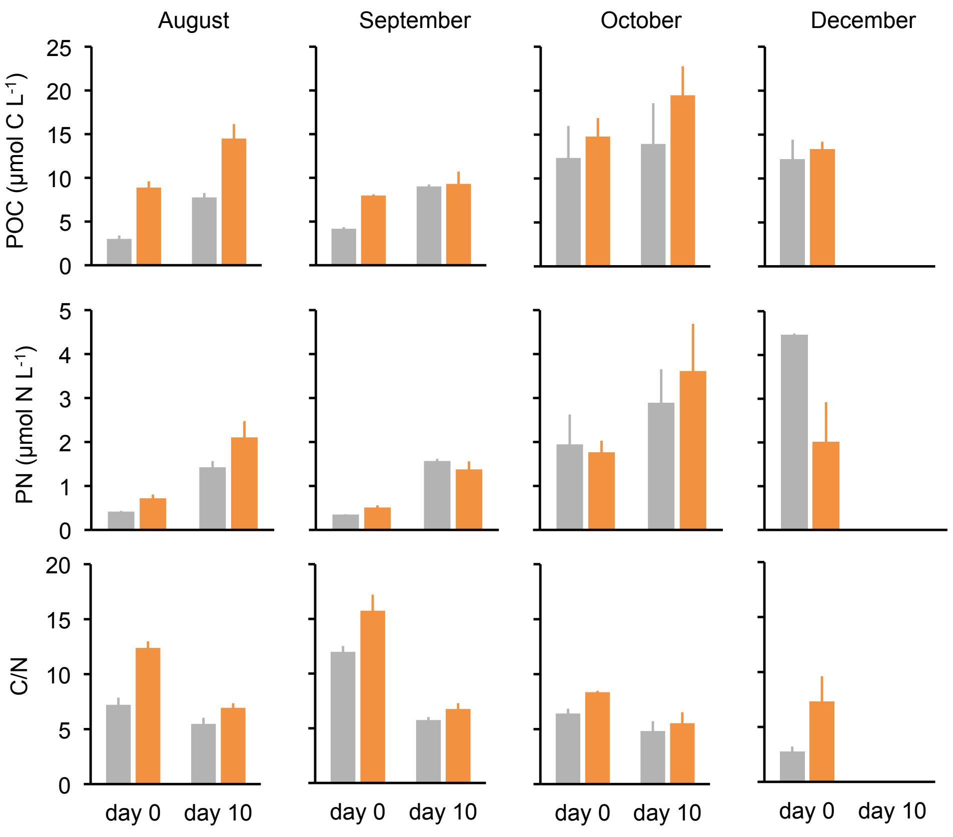

Supplemental Figure 2. Particulate organic carbon (POC), particulate nitrogen (PN), and the C/N ratio of particulate material in mesocosm experiments (average ± standard deviation, n=3). The gray bars are controls and the orange bars are DOM+ treatments. The data from day 10, December were lost due to an instrument malfunction. The C/N ratio of day 0, December controls is very low, suggesting that this particular POC/PN sample may have been comprimised. The POC in DOM+ treatments in August and September is somewhat higher than controls, indicating incomplete dissolution of added DOM. The C/N ratio in DOM+ was higher in August and September, reflecting the higher C/N ratio of added DOM+. This pattern was not evident in October or December, perhaps due to higher POC in the starting seawater. The C/N ratio decreased during incubations, indicating preferential growth of bacteria.

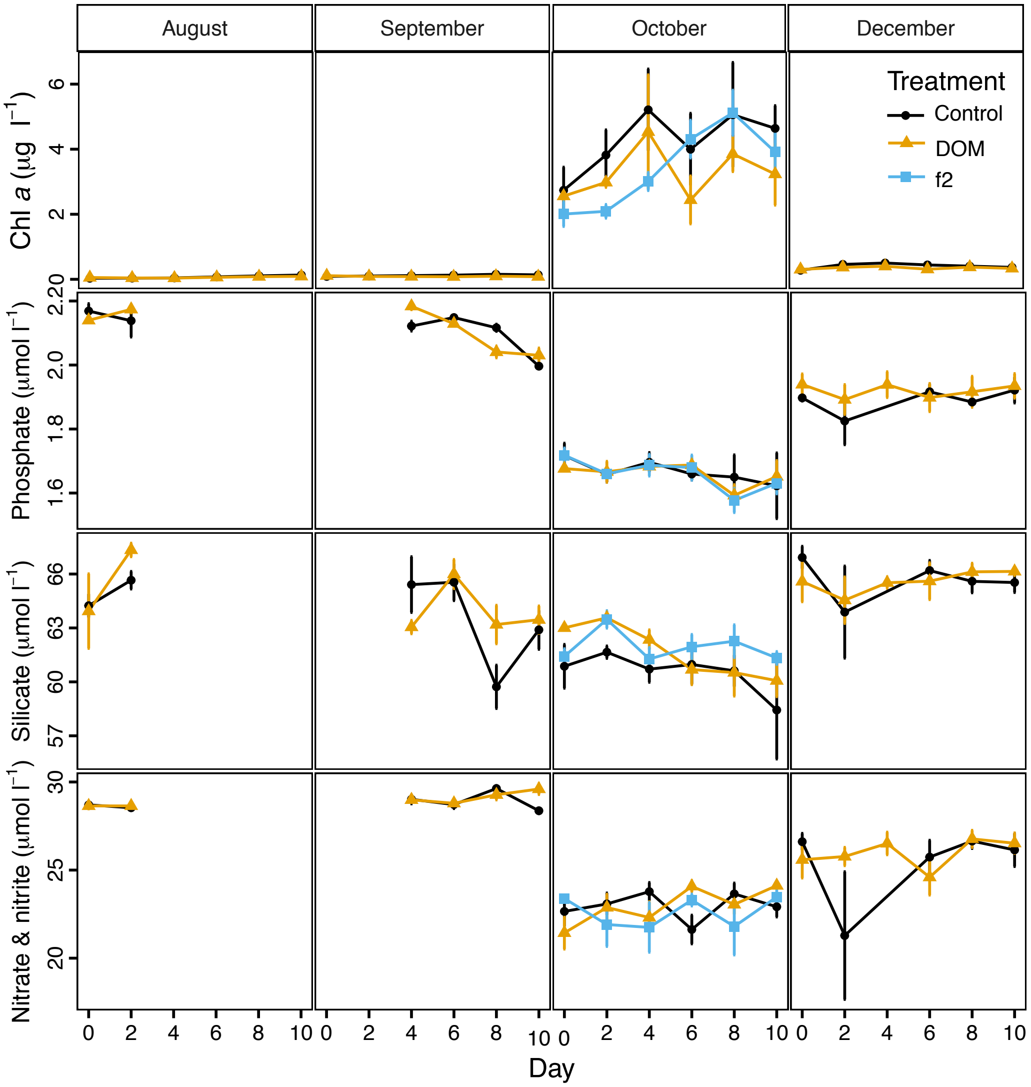

Supplemental Figure 3. Chlorophyll *a* and inorganic nutrient (phosphate, silicate, and nitrate/nitrite) concentrations over the course of four experiments (average ± standard deviation, n=3). Missing data were not available due to loss of sample vials.

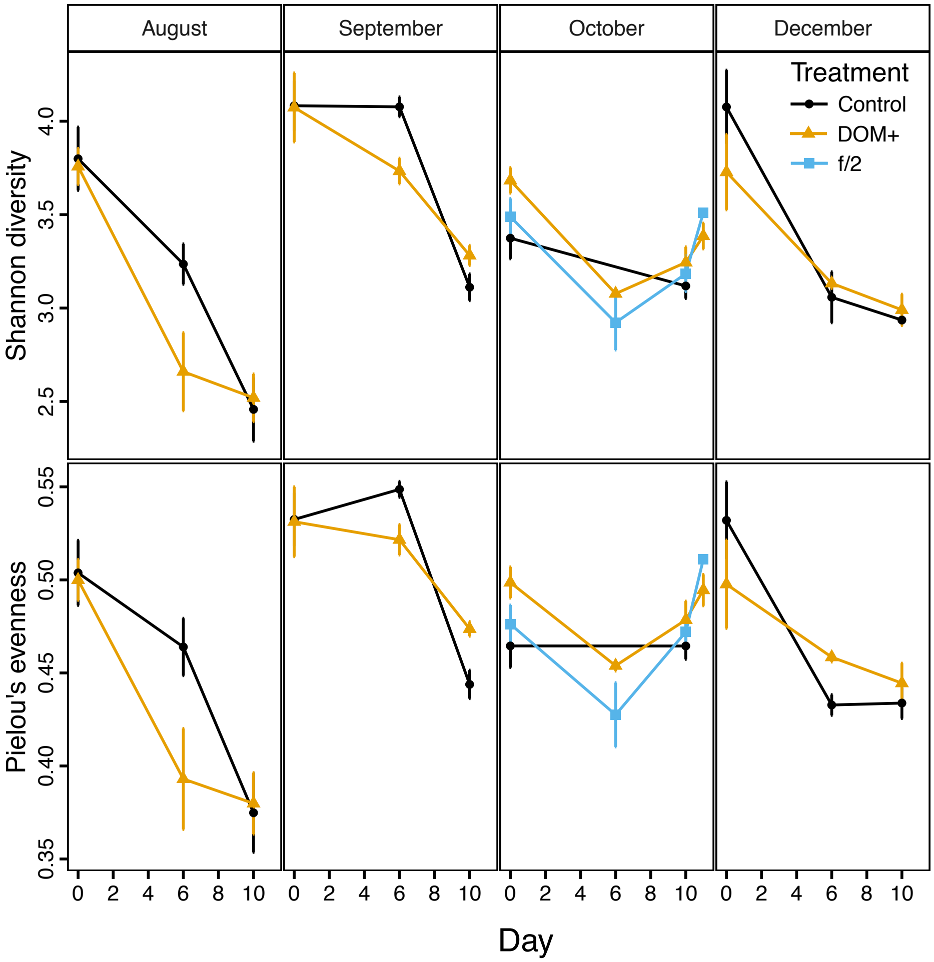

Supplemental Figure 4. Alpha diversity metrics (Shannon diversity and Pielou's evenness) over the course of four experiments (average ± standard deviation, n=3).

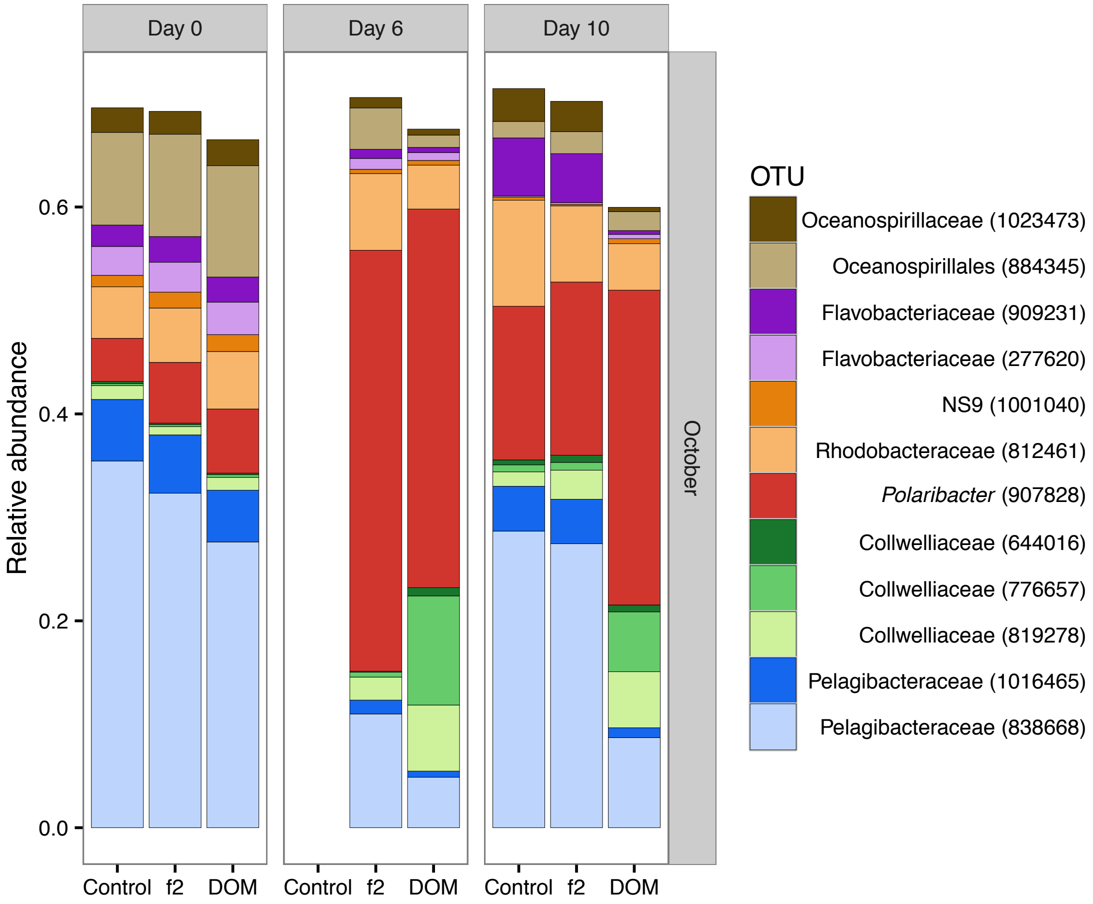

Supplemental Figure 5. Comparison of bacterial community composition between control, f/2, and DOM addition in the October experiment, based on 12 most abundant OTUs. The control and f/2 treatment had similar bacterial community composition on day 10. The comparison on day 6 was not possible, as adequate Illumina-barcoded PCRs of control/day 6 samples were not successful (all other samples were successful). As f/2 was indistinguishable from controls in other respects (bacterial abundance, production, diversity) the f/2 samples were used in place of controls in October, day 6 in further analyses of overall bacterial community composition (Figure 3 and 4).

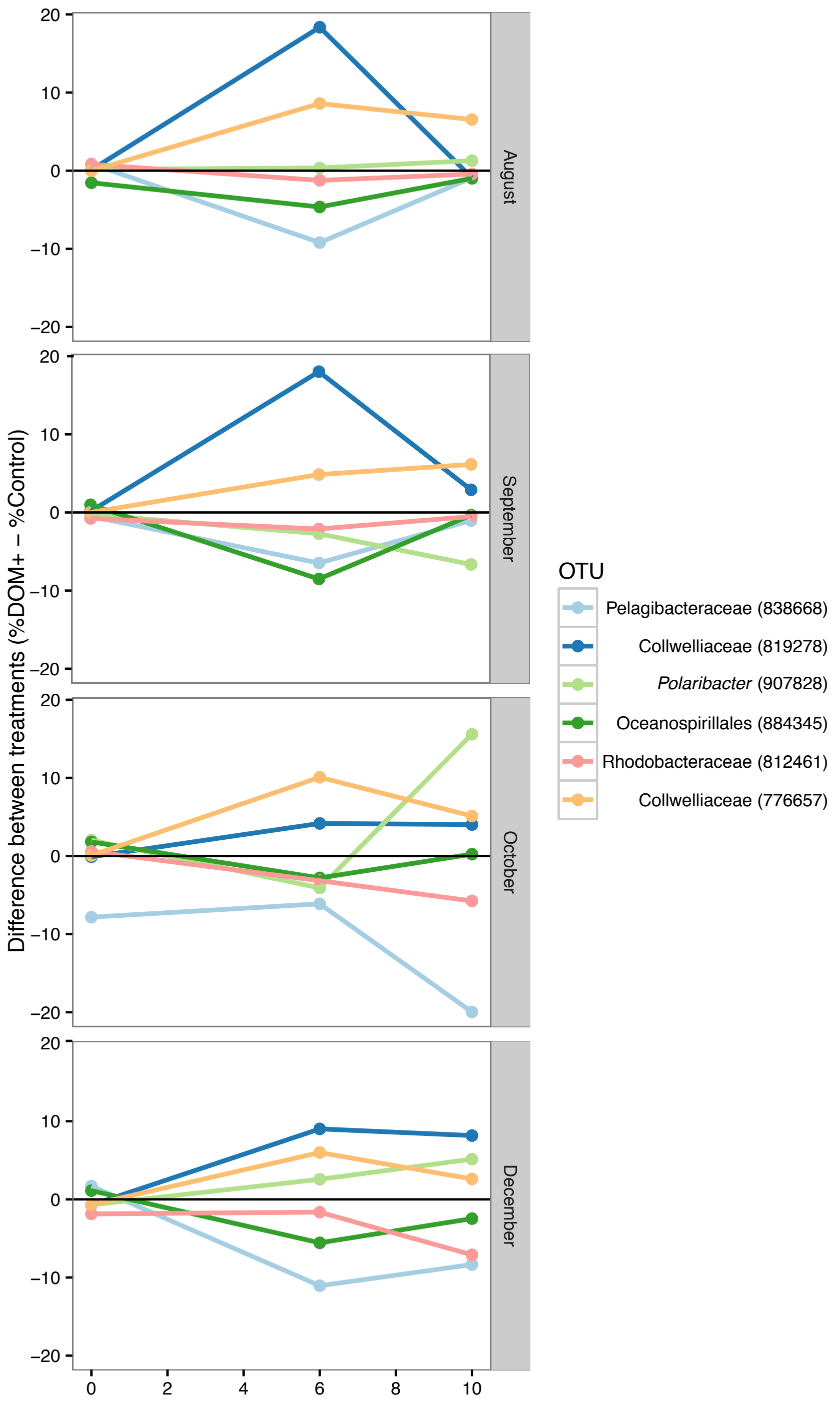

Supplemental Figure 6. Difference in OTU relative abundance (%) between the DOM-amended and control mesocosms. Out of top 12 OTUs (by mean relative abundance), only OTUs showing more than ±5% change are plotted.
